# Non-Hebbian spike-timing-dependent plasticity in cerebellar circuits

**DOI:** 10.3389/fncir.2012.00124

**Published:** 2013-01-11

**Authors:** Claire Piochon, Peter Kruskal, Jason MacLean, Christian Hansel

**Affiliations:** Department of Neurobiology, University of ChicagoChicago, IL, USA

**Keywords:** calcium, climbing fiber, dendrite, long-term depression, long-term potentiation, parallel fiber, Purkinje cell, pyramidal cell

## Abstract

Spike-timing-dependent plasticity (STDP) provides a cellular implementation of the Hebb postulate, which states that synapses, whose activity repeatedly drives action potential firing in target cells, are potentiated. At glutamatergic synapses onto hippocampal and neocortical pyramidal cells, synaptic activation followed by spike firing in the target cell causes long-term potentiation (LTP)—as predicted by Hebb—whereas excitatory postsynaptic potentials (EPSPs) evoked after a spike elicit long-term depression (LTD)—a phenomenon that was not specifically addressed by Hebb. In both instances the action potential in the postsynaptic target neuron is an instructive signal that is capable of supporting synaptic plasticity. STDP generally relies on the propagation of Na^+^ action potentials that are initiated in the axon hillhock back into the dendrite, where they cause depolarization and boost local calcium influx. However, recent studies in CA1 hippocampal pyramidal neurons have suggested that local calcium spikes might provide a more efficient trigger for LTP induction than backpropagating action potentials. Dendritic calcium spikes also play a role in an entirely different type of STDP that can be observed in cerebellar Purkinje cells. These neurons lack backpropagating Na^+^ spikes. Instead, plasticity at parallel fiber (PF) to Purkinje cell synapses depends on the relative timing of PF-EPSPs and activation of the glutamatergic climbing fiber (CF) input that causes dendritic calcium spikes. Thus, the instructive signal in this system is externalized. Importantly when EPSPs are elicited before CF activity, PF-LTD is induced rather than LTP. Thus, STDP in the cerebellum follows a timing rule that is opposite to its hippocampal/neocortical counterparts. Regardless, a common motif in plasticity is that LTD/LTP induction depends on the relative timing of synaptic activity and regenerative dendritic spikes which are driven by the instructive signal.

## Introduction

Hebb's postulate on synaptic modifications, which was formulated in 1949 in his book “*The Organization of Behavior*,” has laid the foundation for subsequent experimental work on memory storage by neuronal assemblies (Hebb, 1949):

“*When an axon of cell A is near enough to excite a cell B and repeatedly or persistently takes part in firing it, some growth process or metabolic change takes place in one or both cells such that A's efficiency, as one of the cells firing B, is increased*.”

A more popular version of this rule—assigned to neurobiologist Carla Shatz—says “neurons that fire together wire together.” The discovery of long-term potentiation (LTP) in 1973 demonstrated that synaptic connections can indeed be strengthened in a use-dependent way, thus reflecting a key prediction of the Hebb postulate (Bliss and Lømo, 1973). LTP is now widely regarded as a potentiation mechanism involved in circuit development and adult learning. However, for more than 20 years, researchers did not dissociate the relative roles of synaptic input and action potential generation in the postsynaptic neuron in the induction of LTP (see Linden, 1999). The implication inherent to Hebb's postulate is that excitatory synapses that contribute to the initiation of action potentials in the target cell will be strengthened. This component of the Hebb rule was demonstrated by spike-timing-dependent plasticity (STDP) studies, in which the relative timing of presynaptic activity and postsynaptic spike firing determines the direction and amplitude of synaptic weight change. Excitatory postsynaptic potentials (EPSPs) preceding postsynaptic action potentials within a time window of up to tens of milliseconds cause LTP, while activation in the reverse order induces long-term depression (LTD) (Markram et al., 1997; Bi and Poo, 1998; Debanne et al., 1998). While Hebb did not explicitly discuss the weakening of synapses in his hypothesis, LTD was suggested in a complementary statement by Stent (Stent, 1973) based on studies by Hubel and Wiesel examining plasticity during the critical period in visual cortex (Hubel and Wiesel, 1965; Wiesel and Hubel, 1965). STDP has generated immense interest as a plasticity mechanism that not only obeys Hebb's rule, but also reconciled LTP studies with a renewed interest in temporal coding (König et al., 1996).

We will begin this review with a description of key features of STDP as observed in hippocampal and neocortical pyramidal cells. Then, we will present recent observations that in CA1 hippocampal pyramidal cells LTP is more sensitive to local dendritic spikes than to backpropagating action potentials that originate in the axon hillhock (Golding et al., 2002). We will discuss these findings in an attempt to reach a general assessment of the role of dendritic spikes in forms of plasticity that depend on the detection of temporal order. There are more variations on the STDP theme: in cerebellum-like structures, such as the dorsal cochlear nucleus (DCN) or the electrosensory lobe (ELL) of mormyrid electric fish, anti-Hebbian STDP has been described, in which EPSPs followed by spikes induce LTD, and activation in the reverse order leads to LTP (Bell et al., 1997; Tzounopoulos et al., 2004). In the cerebellum itself, the available data also point toward an STDP rule with anti-Hebbian timing requirements (Wang et al., 2000). However, there are no regenerative Na^+^ spikes in Purkinje cell dendrites (Stuart and Häusser, 1994; Ohtsuki et al., 2012), and the direction of synaptic gain change at parallel fiber (PF) to Purkinje cell synapses depends on the co-activation of the climbing fiber (CF) input instead (Coesmans et al., 2004). CF activation causes two types of spikes that remain locally restricted: complex spikes in the soma and calcium spikes in the dendrite (Schmolesky et al., 2002; Davie et al., 2008). We will suggest that backpropagating action potentials provide an instructive plasticity signal in the neocortex and hippocampus, and that a similar function is served by the temporal correlation between local dendritic calcium spikes and synaptic activity in Purkinje cells. Thus, cerebellar plasticity is timing-dependent, but does not depend on somatic spike output and is thus non-Hebbian in nature.

## STDP and the backpropagation of somatic action potentials into dendrites

Hebbian plasticity requires that activity at impinging synaptic inputs is paired with an instructive signal in the postsynaptic target neuron. This role can be served by the occurrence of an appropriately timed action potential, which propagates from the initial segment “back” into the dendrites. The discovery of action potential backpropagation into the dendrites was thus a prerequisite for an initial mechanistic description of Hebbian-style STDP. To demonstrate that action potentials are initiated close to the soma and actively invade the dendrites, Stuart, and Sakmann performed somato-dendritic double-patch recordings from layer V pyramidal neurons. They observed that (a) action potentials can be recorded in the dendrites after injection of depolarizing current pulses or synaptic stimulation, and (b) that action potentials are initiated in the axon hillock regardless of whether these action potentials were evoked by somatic or dendritic current injection, or by synaptic stimulation (Stuart and Sakmann, 1994). In summary, these results indicate that action potentials in these neurons are initiated close to the soma, and subsequently backpropagate into the dendrites. Similar observations were made in CA1 hippocampal pyramidal neurons (Spruston et al., 1995).

**Figure 1:**
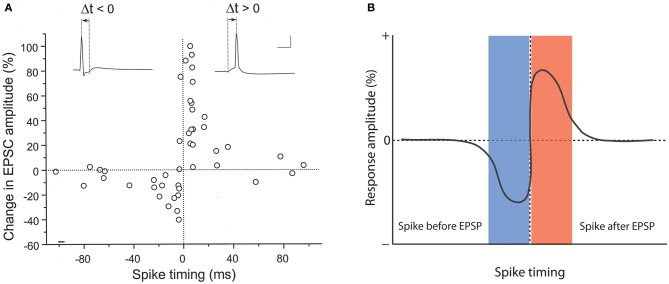
**Hebbian-style STDP in hippocampal neurons. (A)** Temporal order for the induction of LTP and LTD at glutamatergic synapses onto cultured rat hippocampal neurons. The change in EPSC amplitudes measured at 20–30 min after tetanization (60 stimuli at 1 Hz) is plotted against spike timing. Spike timing is defined by the time interval (Δ*t*) between the onset of the EPSP and the spike peak (see traces on top). Scale bars: 50 mV and 10 ms. **(B)** Model scheme for Hebbian STDP. LTD results when the spikes precede the EPSPs, whereas LTP is induced when the EPSPs are evoked before spike onset. **(A)** is taken from Bi and Poo (1998). Copyright 1998 by the Society for Neuroscience.

A role for backpropagating action potentials in plasticity was demonstrated a few years later. It was shown that in CA1 hippocampal pyramidal neurons pairing of subthreshold EPSPs with backpropagating action potentials causes LTP, and that the potentiation did not occur when these two stimuli were applied in isolation, or when spike backpropagation was blocked with local application of tetrodotoxin (TTX; Magee and Johnston, 1997). In a back-to-back paper in the same issue of *Science*, it was demonstrated using dual patch-clamp recordings that pairing of EPSPs with postsynaptic action potentials promotes LTP at synapses between connected layer 5 pyramidal neurons (Markram et al., 1997). This study also looked at the timing of pre- and postsynaptic activity in more detail, and found that LTP is induced when EPSPs precede the action potentials by 10 ms, but that application of these stimuli in reverse order results in LTD. Longer intervals (100 ms) neither elicit LTP nor LTD (Markram et al., 1997). Similarly narrow timing windows (≤20 ms) were found in STDP studied in hippocampal cultures/slice cultures (Bi and Poo, 1998; Debanne et al., 1998). Figure 1A shows original data from the study by Bi and Poo (1998), and illustrate that LTP results either from coincident occurrence of EPSPs and action potentials (0 ms latency), or from EPSPs followed by an action potential (positive latency), whereas LTD is observed when the spike precedes the EPSP (negative latency, see Figure 1B for a model scheme). Backpropagating action potentials evoke calcium transients in the dendrites that result from the activation of voltage-dependent calcium channels (Markram et al., 1995). The amplitude of spine calcium transients evoked by paired activation of EPSPs and action potentials depends on the temporal order. Calcium signals are larger when EPSPs precede action potentials by latencies of less than 50 ms and that calcium influx is less when the sequence is reversed (Koester and Sakmann, 1998; see also Graupner and Brunel, 2007). These findings are in line with the idea that LTP induction has a higher calcium threshold than LTD induction (Bienenstock et al., 1982; Bear et al., 1987; Hansel et al., 1997). This timing between pairings is not sufficient by itself. Packets of multiple pairings with this temporal structure are needed to provide sufficient depolarization, but lower frequency STDP pairings can also be effective given additional somatic depolarization (Sjöström et al., 2001). Further, given a burst of postsynaptic action potential firing paired with a single presynaptic action potential, the direction and extent of plasticity depends on the timing of dendritic calcium transients with the presynaptic spike (Zilberter et al., 2009). It should be noted, however, that the calcium transient amplitude is likely not the only factor involved. For example, it has been shown that the potentiation in STDP-style protocols is NMDA receptor-dependent, while LTD requires the activation of metabotropic glutamate (mGluR) receptors, suggesting that two different calcium sensors downstream of these receptors might regulate LTP and LTD induction (Nevian and Sakmann, 2006). Thus, the localization and specific activation/inactivation conditions of these calcium sensors are likely to influence the calcium signaling requirements as well. Regardless of the underlying details, it seems fair to say that local depolarization events and calcium transients serve key functions in controlling the LTP/LTD balance. But which dendritic activity patterns evoke the appropriate calcium transients under physiological conditions? It has been argued that local dendritic spikes, rather than backpropagating Na^+^ spikes may be instrumental for plasticity control (see Lisman and Spruston, 2010). This challenge to the classic STDP model is based on the observation that (a) backpropagating action potentials typically do not invade distal dendrites, and thus STDP may not be a general plasticity mechanism, (b) LTP can be induced in the absence of Na^+^ spikes, and (c) local depolarization can be more efficient in triggering LTP than backpropagating action potentials (Golding et al., 2002; Hardie and Spruston, 2009). This latter effect might result from the fact that local dendritic events, such as calcium spikes or AMPA/NMDA receptor-mediated responses provide a more prolonged depolarization than fast Na^+^ spikes (Lisman and Spruston, 2010). It is, however, conceivable that Na^+^ spikes, under conditions where they contribute to plasticity, facilitate the initiation of local calcium spikes, and that thus STDP is a physiologically relevant model for plasticity, but acts locally through dendritic calcium spikes. From a mechanistic point of view, these local calcium spikes can present an instructive signal whether or not their occurrence is facilitated by Na^+^ spike backpropagation (Hardie and Spruston, 2009).

## Variations of spike-timing-dependent plasticity: anti-hebbian STDP

**Figure 2:**
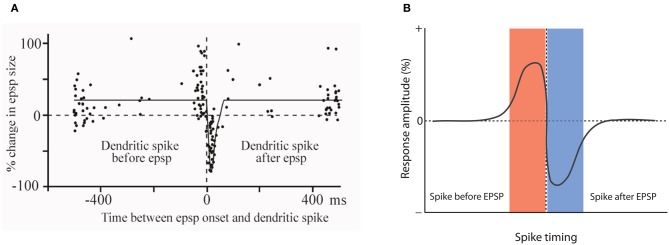
**Anti-Hebbian STDP in a cerebellum-like structure. (A)** Temporal order for the induction of LTP and LTD at parallel fiber synapses onto Purkinje-like cells in the electrosensory lobe of the mormyrid fish *Gnathonemus petersii*. Changes in EPSP amplitudes are plotted against the delay between EPSP onset and the broad spike peak during the pairing period (360 stimuli at 1 Hz). **(B)** Model scheme for anti-Hebbian STDP. LTP is induced when the spike is initiated before an EPSP is evoked. Stimulation in the reverse order (EPSP-spike) results in LTD. Note that this figure panel shows an idealized model of anti-Hebbian STDP and differs from the experimentally obtained data presented in **(A)**, in which potentiation is also seen with intervals >400 ms. **(A)** is modified from Bell et al. (1997) with permission from Macmillan Publishers Ltd: Nature, copyright 1997.

One of the very first reports of STDP did not result from recordings from hippocampal or neocortical pyramidal neurons, but from medium ganglion (MG) cells of the ELL of the mormyrid electric fish *Gnathonemus petersii* (Bell et al., 1997). The ELL is a cerebellum-like structure, and MG cells are GABAergic neurons that are described as Purkinje-like cells—they receive glutamatergic PF synapses, but lack the CF input that is characteristic for cerebellar Purkinje cells. In these Purkinje-like neurons, pairing of a PF-EPSP with a postsynaptic spike results in LTD if the spike follows the EPSP onset within 60 ms. In contrast, LTP is induced when the spikes are delivered outside this time window, or PF-EPSPs are evoked at 1 Hz in the absence of spikes (Bell et al., 1997; Han et al., 2000). Thus, STDP in this cerebellum-like structure follows an anti-Hebbian temporal order (Figure 2). The available data support the notion that this type of STDP is under control of the spike output of the postsynaptic target cell. The spikes that were evoked in these experiments by somatic current injection are so-called “broad spikes” that are TTX-sensitive (Bell et al., 1997), and are initiated in the soma/proximal dendrite, from where they propagate into the apical dendrite (Gomez et al., 2005; Engelmann et al., 2008). Broad spikes certainly differ from fast action potentials that are capable of producing cortical STDP—broad spikes are 8–15 ms wide and only reach amplitudes in the range of 40–60 mV (Bell et al., 1997). Still, these spikes are at least partially mediated by voltage-gated Na^+^ influx and are initiated in or near the soma, providing a signal that reflects the electrical output of MG cells. Thus, it seems fair to state that STDP in the ELL is anti-Hebbian with regard to the temporal order controlling LTP and LTD induction, but nevertheless falls into the category of Hebbian-style learning rules, because of the critical involvement of spike backpropagation into the dendrites. This type of anti-Hebbian STDP is not restricted to non-mammalian vertebrates, but has also been described in a mammalian cerebellum-like structure, the DCN, which is a brainstem region that is part of the auditory system. In cartwheel cells, which are inhibitory interneurons that resemble MG cells in the fish ELL, activation of EPSPs by PF stimulation leads to LTD if the EPSPs are followed after 5 ms by spike activity. No synaptic change results from activation in the reverse order (Tzounopoulos et al., 2004). In cartwheel cells, somatic depolarization leads to simple spike and/or complex spike firing, and is believed to trigger dendritic calcium spikes. The depression resulting from EPSP-spike sequences is presynaptically expressed and requires retrograde cannabinoid signaling (Tzounopoulos et al., 2007). Similarly, LTD induced by spike-EPSP sequences in layer 5 pyramidal neurons has been shown to require the activation of presynaptic CB1 receptors (Sjöström et al., 2003). Thus, this signaling mechanism is not restricted to anti-Hebbian STDP. We will discuss below that while anti-Hebbian STDP has been described in most detail in cerebellum-like structures, a form of STDP with anti-Hebbian (and non-Hebbian) components also plays a role in the cerebellum itself. Moreover, modeling studies have suggested that in CA1 hippocampal pyramidal neurons, anti-Hebbian STDP could function to equalize synaptic weights along the axis of the apical dendrite (Rumsey and Abbott, 2006). Due to the scope of this review, however, we will not discuss these modeling studies in detail.

## Non-hebbian STDP in the cerebellum

Surprisingly, STDP has not been studied in as much detail in the cerebellum proper. This might be due to the fact that in the cerebellum, LTP has been discovered later than in most other brain areas. While a presynaptic form of LTP has been described in 1996 (Salin et al., 1996), postsynaptic LTP—a potential reversal mechanism for the postsynaptically expressed LTD—has only been documented in 2002 (Lev-Ram et al., 2002). Nevertheless, sufficient data are available to draw some conclusions. LTD at PF synapses onto Purkinje cells results from co-activity of the PF and the CF input (Ito et al., 1982), during which the CF triggers complex spikes that can be recorded in the soma (for review, see Schmolesky et al., 2002). The first study that looked at timing requirements reported that LTD was induced best when CF stimulation (and complex spike activity) preceded PF activation with an interval of less than 250 ms (Ekerot and Kano, 1989). However, this report was inconclusive as (a) LTD was monitored with extracellular recordings of simple spikes—a measure that does not directly reflect synaptic plasticity, and (b) LTD was also observed when PF activity preceded CF activity by 5–20 ms. Subsequent studies found that LTD is most efficiently induced when PF stimulation precedes complex spike activity by 50–250 ms (Chen and Thompson, 1995; Wang et al., 2000; Safo and Regehr, 2008). Chen and Thompson showed that 600 pairings of PF and CF activity caused LTD independent of the timing interval. However, when using only 100 pairings, LTD is more sensitive to timing requirements: LTD was induced best when PF stimulation preceded CF activity by 250 ms, a depression that did not reach statistical significance resulted from PF+CF co-activation with 125 ms or 0 ms intervals, and no change was observed when CF stimulation preceded PF activity by 250 ms (Chen and Thompson, 1995). Similarly, Wang et al. showed that LTD is induced when PF stimulation precedes complex spike activity by 150 ms or when the two inputs are simultaneously activated, but LTD does not result from co-activation using a 500 ms interval, or a reversed activation sequence (150/500 ms interval). Interestingly, although not discussed by the authors, CF-PF stimulation with an interval of 150 ms results in a weak potentiation that lasts about 20 min (Wang et al., 2000). Safo and Regehr obtained similar results, but also noted a depression when CF activity preceded PF activity by 50 ms (Safo and Regehr, 2008). However, since more pronounced LTD was induced by PF stimulation first (50 and 150 ms), these data generally seem to confirm the observations made by the other two groups. These studies point toward an anti-Hebbian STDP mechanism in the cerebellum, similar to the anti-Hebbian STDP described in cerebellum-like structures (but note that in cerebellum-like structures the timing intervals seem to be shorter). In the light of the discrepancies in the literature, we would like to point out that in our hands 100 Hz PF burst stimulation followed after 120 ms by CF activity causes LTD (Piochon et al., 2010), which is in line with the observations by Chen and Thompson (1995) and Wang et al. (2000). At the single spine level, evoked calcium transients are largest when the PF input is activated before CF stimulation (Wang et al., 2000). This observation might explain why this temporal sequence is optimal for LTD induction, since at these cerebellar synapses LTD has a higher calcium threshold for induction than LTP (Coesmans et al., 2004).

Remarkably, such a requirement for specific temporal order and similar activation sequences can also be observed in behavioral learning. One example is the need for temporal specificity in associative eyeblink conditioning—a form of motor learning that involves the cerebellum: the optimal interval between application of a tone (conditioned stimulus; conveyed by the PF input) and an air puff application to the eye (unconditioned stimulus; conveyed by the CF input) is between 200–400 ms, thus offering a rare opportunity to relate timing intervals that were observed in *in vitro* and *in vivo* learning studies, respectively (Thompson and Krupa, 1994). In line with these results, in gain adaptation of the vestibulo-ocular reflex (VOR), another type of learning mediated by the cerebellum, CF activity needs to follow PF activity by 100 ms (Raymond and Lisberger, 1998). These examples illustrate that both LTD induction and forms of behavioral learning require PF activity prior to CF activation for the adaptive change to occur.

Cerebellar plasticity depends on the relative timing between the activation of PF synapses and the occurrence of CF-evoked complex spikes. As a result it appears that in STDP an instructive signal for the strengthening or weakening of a synapse can arise from a number of sources: a backpropagating action potential or a synaptically evoked calcium spike depending on the structure or system. However, cerebellar STDP differs from STDP at hippocampal/neocortical synapses, and also from STDP in cerebellum-like structures, in that it does not depend on the axonal spike output. In cerebellar Purkinje cells—in contrast to pyramidal cells and Purkinje-like cells—Na^+^ action potentials that are elicited in the axon hillhock do not actively backpropagate, but rather spread passively into the dendrites (Stuart and Häusser, 1994; Ohtsuki et al., 2012). Thus, the dendrite does not receive feedback information on whether action potentials were fired or not, which is a key component of Hebbian plasticity (but note that dendritic calcium transients can vary in amplitude depending on whether the cell is in an up or down state; Kitamura and Häusser, 2011). Rather, bidirectional PF plasticity depends on the relative timing with activity of the heterosynaptic CF input, which provides an externalized instructive signal. Interestingly, the somatically recorded complex spike does not seem to play a role in cerebellar STDP: double-patch recordings from the soma and dendrite of Purkinje cells have shown that the classic complex spike waveform only occurs in the soma, and that CF activation results in all-or-none EPSPs in the dendrites instead, which can be associated with local spike activity (Davie et al., 2008; Ohtsuki et al., 2009, 2012). As these dendritic spikes typically do not cause additional spikes near the soma, it seems that CF activity evokes two types of spikes in the soma and dendrites that occur independently from each other. Thus, STDP at cerebellar PF to Purkinje cell synapses is anti-Hebbian with regard to the optimal temporal order of synaptic activation and the occurrence of local dendritic spikes, but is non-Hebbian with regard to the lack of action potential backpropagation and thus information on the neuron's spike firing output.

As outlined above, PF plasticity in both the cerebellum proper and in cerebellum-like structures follows an anti-Hebbian STDP rule, but in contrast to Purkinje cells in the mammalian cerebellum, Purkinje-like cells in cerebellum-like structures such as the mormyrid ELL show action potential backpropagation, which may be involved in STDP. To reconcile these observations we review plasticity mechanisms in the mormyrid cerebellum proper. Neither STDP nor action potential backpropagation have been systematically studied in cerebellar Purkinje cells of mormyrid fish. However, it has been shown that PF-LTD results from PF activation followed after 20–50 ms by CF stimulation, pointing toward an anti-Hebbian STDP mechanism (Han et al., 2007). The role of the axonal spike output remains unclear. CF activation does not result in complex spikes, but in all-or-none EPSPs. Moreover, Na^+^ action potentials recorded from the somata of mormyrid Purkinje cells have unusually small amplitudes, typically not exceeding 30 mV (de Ruiter et al., 2006). It seems unlikely that these reduced Na^+^ spikes backpropagate, although mormyrid Purkinje cells express the Na^+^ channel α subunits Na_v_1.1, Na_v_1.2, and Na_v_1.6 in their dendrites (de Ruiter et al., 2006). On the other hand, somatic depolarization or CF stimulation can evoke broad spikes that can be recorded in the somata of mormyrid Purkinje cells and are associated with dendritic calcium transients that are at least partially mediated by P/Q-type voltage-gated calcium channels (Han et al., 2007). In contrast to their mammalian counterparts, mormyrid CFs only contact very proximal parts of the dendrite (“horizontal dendrite”) and do not invade the molecular layer. Thus, activation in or close to the soma can evoke calcium spikes (broad spikes) in the dendrites. The relevance of these broad spikes for plasticity is currently not understood: pairing PF activation with broad spikes evoked by CF stimulation causes LTD, but when the broad spikes are triggered by somatic injection of depolarizing currents LTP is induced instead (Han et al., 2007). Further studies are needed to determine under which conditions broad spike activity promotes LTD and LTP, respectively, and to assess the role of the axonal spike output in STDP in cerebellar Purkinje cells of mormyrid fish.

**Figure 3:**
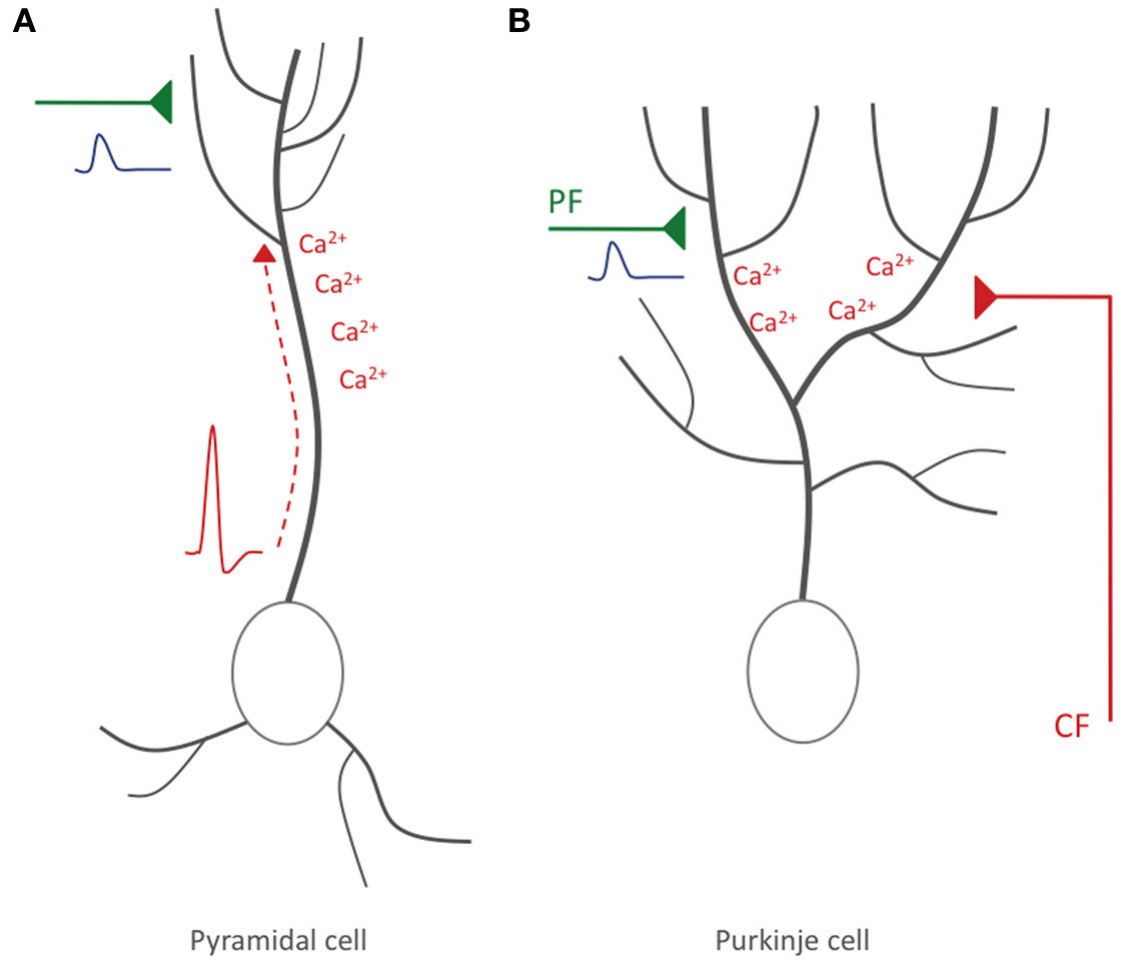
**Hebbian-style and non-Hebbian STDP. (A)** Hebbian STDP in hippocampal and neocortical pyramidal cells. Action potentials are elicited near the soma and backpropagate into the dendrite, where the accompanying depolarization leads to calcium influx (red). The timing relative to incoming EPSPs (blue) evoked at glutamatergic synaptic inputs (green) determines whether LTP or LTD is induced. **(B)** Non-Hebbian STDP in cerebellar Purkinje cells. Here, somatic/axonal action potentials do not actively invade the dendrite. Rather, CF activation causes local dendritic calcium spikes. LTD results if PF-EPSPs precede CF activity. In hippocampal/neocortical circuits this activation sequence (synaptic activity—spike) promotes LTP instead.

In summary, it can be concluded that a form of STDP does exist in cerebellar Purkinje cells, but that there are two important differences to STDP in pyramidal cells: (a) the optimal temporal order of synaptic activation and spike firing is reversed, so that synaptic activity followed by spike activity results in LTD rather than LTP induction, and (b) in mammalian Purkinje cells (and possibly mormyrid Purkinje cells), the instructive signal is externalized, and locally elicited calcium spikes play a key role instead. This latter difference has important functional consequences: in contrast to Hebbian plasticity, what matters is not the timing relative to the axonal spike output, but rather the timing relative to the activity of the CF input, a qualitatively different, heterosynaptic input (Figure 3).

## Was donald hebb wrong?

To answer this question, it needs to be acknowledged first that the famous Hebb postulate—as cited in the introduction—is only a small component within a larger conceptual framework that Hebb presented in his book “The Organization of Behavior.” When using the terms “Hebbian” and “non-Hebbian,” we thus specifically refer to the spike timing mechanism described in the Hebb postulate. Within this framework, the Hebb postulate describes a learning rule for types of neurons, in which action potential backpropagation takes place. By extension, cerebellar STDP can be described as “non-Hebbian” as Purkinje cells lack regenerative backpropagating Na^+^ spikes (Stuart and Häusser, 1994; Ohtsuki et al., 2012).

STDP does exist in the cerebellum, but depends on dendritic calcium spikes instead. Moreover, the temporal order of STDP found in the cerebellum and in cerebellum-like structures is opposite to that expected from an STDP mechanism that follows the Hebb rule (e.g., Bell et al., 1997; Wang et al., 2000). We argue that the Hebb rule remains a widely applicable plasticity concept, but that there are important exceptions and limitations that need to be acknowledged when generalizing.

To understand STDP rules, it is useful to take mechanistic aspects of LTP and LTD induction into consideration. Both forms of plasticity are initiated by local dendritic calcium transients, whose specific features, such as amplitude, localization, and kinetics ultimately determine the polarity of synaptic weight change (for discussion, see Bear et al., 1987; Hansel et al., 1997; Wang et al., 2000; Coesmans et al., 2004; Nevian and Sakmann, 2006). This strict dependence on calcium signaling explains why, for example, LTP in pyramidal cells can be triggered either by local spikes in the dendrites, or by action potential backpropagation: both types of spike activity are associated with local calcium influx (Markram et al., 1995; Golding et al., 2002). At more distal synaptic input locations local calcium spikes may be the most effective means to trigger LTP (Golding et al., 2002; Hardie and Spruston, 2009). However, this observation does not generally exclude a role for Hebbian STDP in plasticity, particularly at more proximal synapses. It remains to be determined which type of spike activity is most relevant under physiological conditions (see also Lisman and Spruston, 2010).

Does the existence of a very different type of STDP (non-Hebbian/reverse temporal order) in the cerebellum challenge the general importance of Hebbian-style plasticity mechanisms? Not necessarily, because cerebellar Purkinje cells have unique features that set them apart from other types of neurons. Of these features, two are particularly relevant for our discussion, because they mark significant differences to hippocampal and neocortical pyramidal cells: first, there is no action potential backpropagation into Purkinje cell dendrites (Stuart and Häusser, 1994; Ohtsuki et al., 2012). As a consequence, the dendrites receive no information on the axonal spike output. However, we suggest that the role of the backpropagating action potential is served by the instructive signal from the CF. Second, Purkinje cells spontaneously fire action potentials (simple spikes) at discharge rates in the range of ~20–80 Hz (Häusser and Clark, 1997), whereas pyramidal cells are almost silent at rest (Margrie et al., 2002). This latter difference is important, because low firing rates allow pyramidal neurons to act as coincidence detectors (König et al., 1996). In contrast, the high firing rates found in Purkinje cells prevent these neurons from using relative spike timing as a relevant measure for processes such as STDP, because the short interval between spikes makes it difficult to distinguish between post- and pre-spike events. This notion holds for simple spikes—that are intrinsically triggered and can result from PF activity—but not for spikes evoked by CF discharges that occur at 1–2 Hz at rest (Simpson et al., 1996). Thus, it does not come as a surprise that STDP at PF synapses onto Purkinje cells is based on timing relative to CF-evoked spike activity (Chen and Thompson, 1995; Wang et al., 2000). A remarkable consequence is that STDP at these cerebellar synapses depends on the relative timing of activity at two qualitatively different, independent synaptic inputs. The externalization of the instructive signal in the cerebellum is very different from the prevalent theme of Hebbian plasticity in neocortex and hippocampus of timing relative to somatic/axonal action potential firing (Figure 3). In the cerebellum, this non-Hebbian form of STDP allows the CF input to assume the role of a teacher and instructor in cerebellar motor learning (Simpson et al., 1996). Cerebellar STDP seems unique in that it depends on specific features of Purkinje cell physiology and of the cerebellar microcircuit. Thus, a more general reading of Hebb's postulate is necessary for it to be applied throughout the central nervous system.

### Conflict of interest statement

The authors declare that the research was conducted in the absence of any commercial or financial relationships that could be construed as a potential conflict of interest.
